# EpiCollect: Linking Smartphones to Web Applications for Epidemiology, Ecology and Community Data Collection

**DOI:** 10.1371/journal.pone.0006968

**Published:** 2009-09-16

**Authors:** David M. Aanensen, Derek M. Huntley, Edward J. Feil, Fada'a al-Own, Brian G. Spratt

**Affiliations:** 1 Department of Infectious Disease Epidemiology, Imperial College London, London, United Kingdom; 2 Centre for Bioinformatics, Imperial College London, London, United Kingdom; 3 Department of Biology and Biochemistry, University of Bath, Bath, United Kingdom; University of Oxford, United Kingdom

## Abstract

**Background:**

Epidemiologists and ecologists often collect data in the field and, on returning to their laboratory, enter their data into a database for further analysis. The recent introduction of mobile phones that utilise the open source Android operating system, and which include (among other features) both GPS and Google Maps, provide new opportunities for developing mobile phone applications, which in conjunction with web applications, allow two-way communication between field workers and their project databases.

**Methodology:**

Here we describe a generic framework, consisting of mobile phone software, EpiCollect, and a web application located within www.spatialepidemiology.net. Data collected by multiple field workers can be submitted by phone, together with GPS data, to a common web database and can be displayed and analysed, along with previously collected data, using Google Maps (or Google Earth). Similarly, data from the web database can be requested and displayed on the mobile phone, again using Google Maps. Data filtering options allow the display of data submitted by the individual field workers or, for example, those data within certain values of a measured variable or a time period.

**Conclusions:**

Data collection frameworks utilising mobile phones with data submission to and from central databases are widely applicable and can give a field worker similar display and analysis tools on their mobile phone that they would have if viewing the data in their laboratory via the web. We demonstrate their utility for epidemiological data collection and display, and briefly discuss their application in ecological and community data collection. Furthermore, such frameworks offer great potential for recruiting ‘citizen scientists’ to contribute data easily to central databases through their mobile phone.

## Introduction

Increasingly, the web is used in biology as a method to collate and analyse data collected by field workers (for example, ecologists or epidemiologists) from multiple locations. A single central database, accessed through a website, can provide the tools for the submission, visualisation and analysis of data collected by many users from many different locations. A good example is provided by the molecular epidemiological databases that have been developed for a number of bacterial and fungal pathogens, where communities of researchers and public health laboratories submit data on strains of individual pathogens, including basic epidemiological information and molecular typing data (for example, http://www.mlst.net) [1], [2]. By linking these databases to Google Maps, the geographic distribution of pathogen genotypes can be displayed and the databases can be explored and analysed conveniently (http://maps.mlst.net).

Central web databases for the collation of data submitted by field workers are also of utility to ecologists, and for mapping distributions of endangered species, and a single web portal can significantly decrease the time between collation and analysis of data collected by multiple users. However, transferring data to these databases (e.g. from field notebooks) can be tedious and in some cases will be subject to transcription errors. Alternatives to paper collection, such as personal digital assistants (PDAs) or standard mobile phones, with subsequent synchronization with a user's laptop or desktop computer, offer attractive opportunities for remote data collection in many areas. For example, EpiSurveyor (www.episurveyor.org) allows the use of standard Nokia mobile phones for the collection of text-based data and has been utilised in resource-poor areas of Africa for many kinds of data collection.

Recently, the availability of a new generation of mobile phones, bracketed under the term ‘smartphones’, offers further novel approaches by the development of software that allows submission and two-way retrieval of data from the field to a central database by mobile phone [3], [4].

Smartphones offer PC-like functionality and web connectivity far superior to traditional mobile phones. Built-in GPS receivers provide the detailed location of the phone, accelerometers can recognise changes in movement, and cameras provide the ability to record static images as well as video. Data networks allow in-built software to access the internet providing web browsing, email and mapping (such as Google Maps) and office documents can be viewed/edited using touch screen keyboards (or hardware keyboards) for textual input.

The versatility of these phones is greatly enhanced by the ability for software developers to produce and deploy their own applications directly on the phones. Google, in conjunction with the Open Handset Alliance (a group of more than 45 technology and mobile phone companies), has recently released the Android operating system for mobile devices (http://www.android.com). Android is a completely open source operating system for, but not restricted to, mobile phones. Android gives software developers the freedom to access all aspects of a smartphone's functionality and software is delivered to phones through the ‘Android Market’, an in-built application that provides a user with a ‘click and install’ method based on browsing a list of all developer-produced software. The first handsets running Android were released in October 2008 and currently four variants are commercially available, the T-Mobile G1 and G2, the Vodafone HTC Magic and the Orange HTC Hero with other manufacturers releasing handsets in the near future.

The increased sophistication of smartphones and developments within web technology, loosely grouped under the heading of ‘Web 2.0’, allow web developers to produce ‘mashups’ of data from different sources, the most well known examples of which involve displaying data in its geographical context using Google Maps [5]. The availability of ‘geographically aware’ mobile phones which can interact with central web databases, visualising data within the geographical context that they were collected, provides the opportunity to develop new data collection, visualisation and analysis strategies for many kinds of biological data, particularly for field workers and epidemiologists.

Here we present a framework for such data collection, and for the two-way communication between field workers and their project databases, which takes advantage of the open source nature of Android (to develop mobile phone software) and open development tools for web applications (Google Maps and Google Earth). A key aspect of this approach is that, as well as providing a way of submitting data by phone to a project database, all data or selected data can be retrieved from the database and viewed and analysed using Google Maps, both via the web and on the mobile phone. In this way the field worker has essentially the same ability to display and analyse data from the project database on their mobile phone as they would have if they were back in their laboratory accessing their database via the web. Our initial examples focus on the use of this framework for the purposes of epidemiological data collection, but EpiCollect has been designed to be generic, and the same principles are applicable for a wide range of projects, some of which are discussed.

## Results and Discussion

### The framework

**Figure 1:**
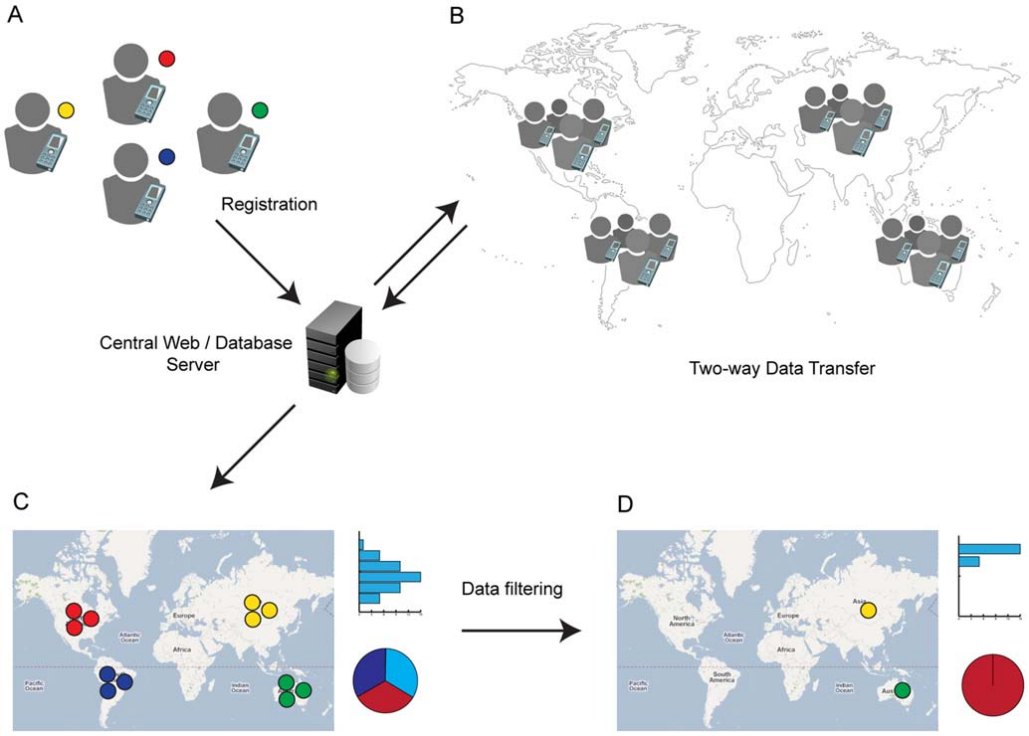
Data collection, collation and visualisation framework using EpiCollect and www.spatialepidemiology.net. See text for details.

The framework consists of two major components. Firstly, we have developed generic software for Android, EpiCollect, which allows multiple data records to be entered and stored on a mobile phone (text variables, GPS position, photo etc.) and sent to a central web database. Secondly, we have developed a web application, located within www.spatialepidemiology.net that, for a specific project, allows the mapping, visualisation and analysis of data submitted to the central database. A schematic of the two-way connectivity between mobile phone and project database obtained using EpiCollect and spatialepidemiology.net is shown in Figure 1.

For a new project, data variables to be collected for each data record (sample) are firstly defined. These variables are then used in three ways. 1) To define the fields in the project-specific central database and the web forms for standard data entry via the project website. 2) To define the interface for text input for each data record within EpiCollect loaded onto a mobile phone. 3) To define the data values or ranges for filtering the data that are displayed at the project website or on the mobile phone.

For each project, the central registration of phones loaded with EpiCollect is undertaken using a curator's interface (Figure 1A), allowing multiple users to submit data centrally to the project database (Figure 1B). The project-specific website provides a public interface that displays all data using Google Maps or Google Earth (Figure 1C). Points on the map can be coloured either by the user (or group of users) who submitted the data, or alternatively, based on a variable submitted to the database. Furthermore, the display of data can be filtered based on the variables collected. For example, filtering allows display of only those samples obtained within a specific range of a variable or within a specific time period, and graphical summaries based on the variables collected are displayed (Figure 1D). Each time a new data record is sent from EpiCollect to the central database a new point appears on the map and the variables become immediately available for filtering. This allows the progress of the project to be monitored in real-time and, furthermore, a central project curator can, while fieldwork is underway, communicate with individual users, or all users, via instant messaging provided by Google Talk.

### EpiCollect

**Figure 2:**
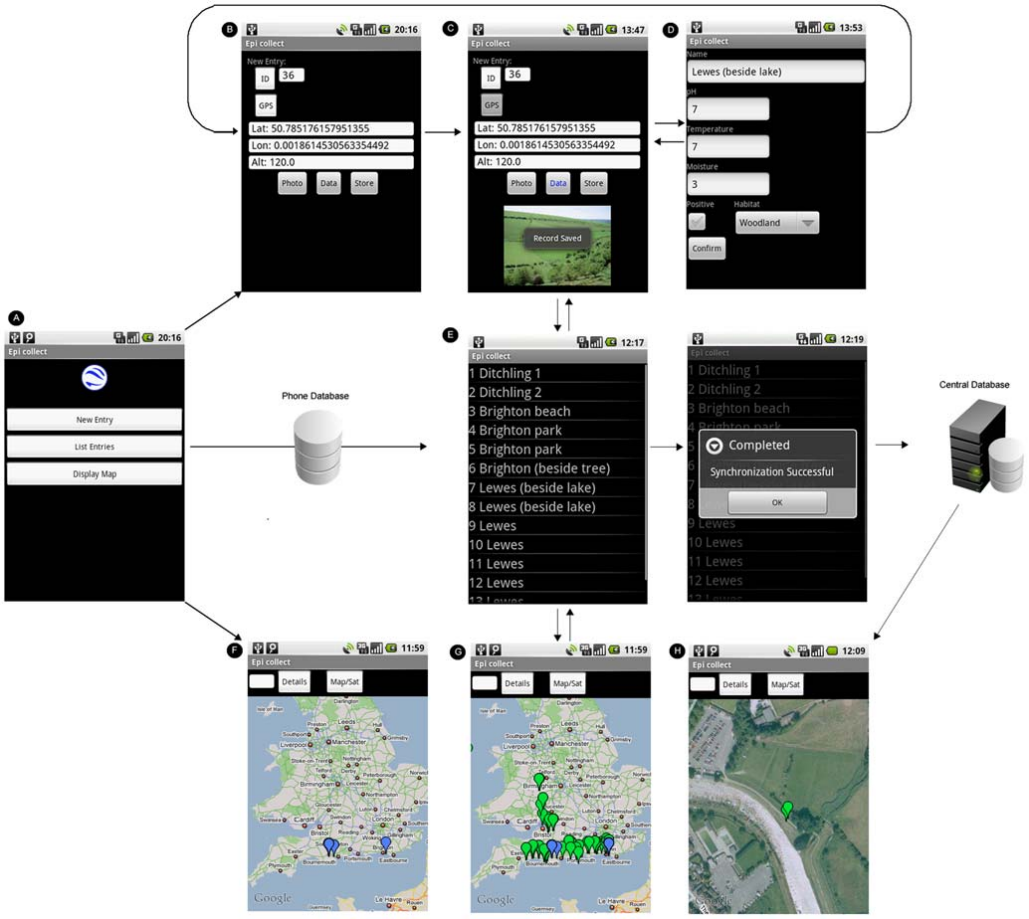
The EpiCollect workflow. See text for details.

When EpiCollect is launched on the mobile phone three options are initially available, ‘New Entry’, ‘List Entries’ and ‘Display Map’ (Figure 2A). Selecting ‘New Entry’ creates a new data record within the phone's on-board database and assigns a unique ID to the record. The latitude, longitude and altitude of the current position of the user is returned from the GPS unit of the phone (Figure 2B). Three new options are now available, ‘Photo’, ‘Data’ and ‘Store’. Selecting photo allows an image to be taken using the phone's camera, which is assigned to the record (Figure 2C). Selecting ‘Data’ displays the data entry screen (Figure 2D), which can contain any standard form field (text fields, list boxes, check boxes etc.). These fields correspond to those created when defining the project database structure. Data can be entered via an on-screen keyboard (using the touch-screen) or via the hardware keyboard should this be part of the phone (e.g. T-Mobile G1). Following data entry, the ‘Confirm’ button returns the view to the Entry screen (Figure 2C). Selecting ‘Store’ saves the current record to the phone's database along with the date and time that the record was created. Subsequently, this process is repeated to create further records.

The ‘List entries’ option allows all records stored within the phone's database to be viewed and amended if necessary. From this screen, data can be synchronised with the central database with confirmation of successful data transfer (Figure 2E). The ‘Display Map’ option allows all records stored locally on the phone to be displayed on a map using the in-built Google Maps application (Figure 2F). Furthermore, records based on a particular variable (or all records) can be retrieved from the central database and displayed on the phone using Google Maps in conjunction with the locally stored records (Figure 2G). The standard click-drag-zoom interface to the map is available on the phone allowing map navigation and can, using the GPS, instantly zoom to the phone's current location (Figure 2H).

### Field Testing

A simple field test of EpiCollect was carried out in which soil samples were taken across Southern England by a single field worker, to obtain isolates of a soil bacterium (*Xenorhabdus nematophila*) that is pathogenic to insect larvae. At each location, a sample of soil was taken, along with a photograph of the area. Data variables were then recorded and entered into EpiCollect including soil pH, temperature and moisture. Data from each sampling location were then submitted via EpiCollect from the phone to the central database located at http://www.spatialepidemiology.net/epicollect/xenorhabdus.

**Figure 3:**
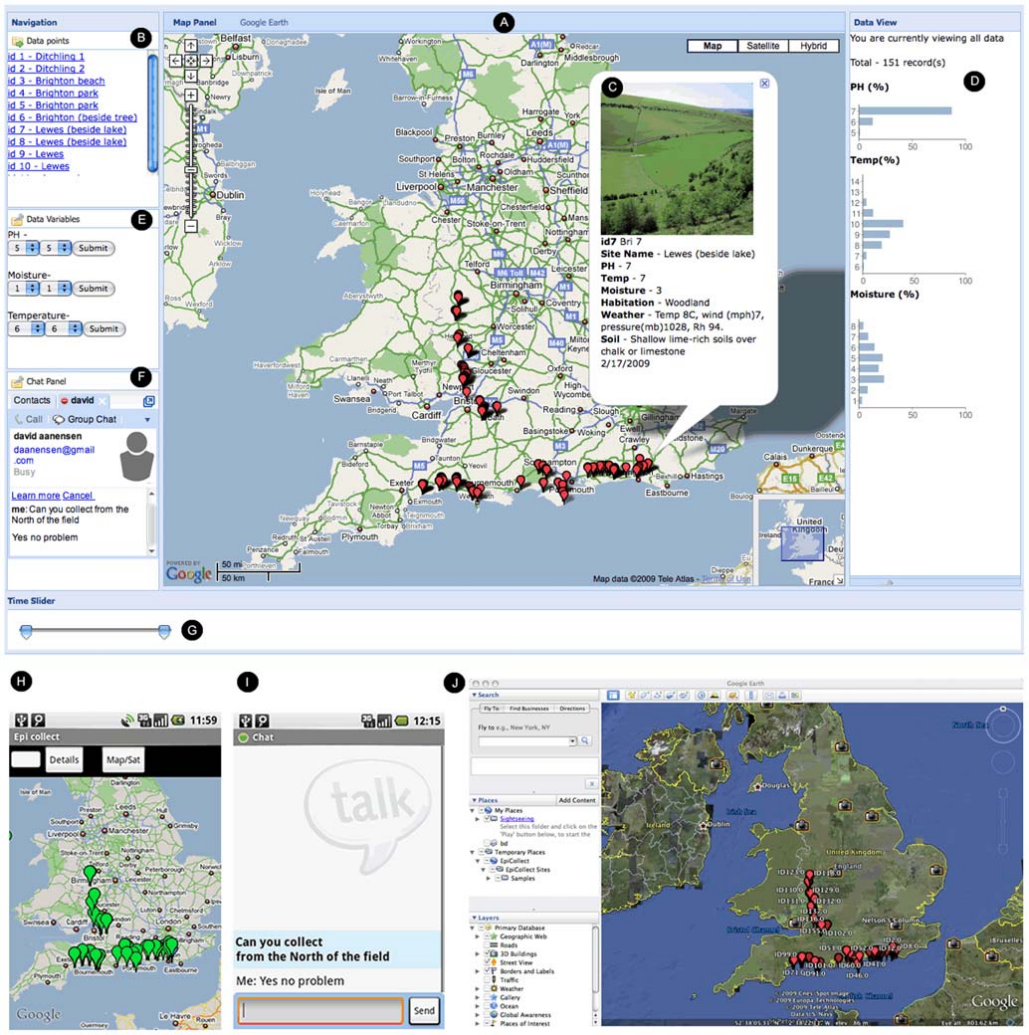
Simple field test of EpiCollect with data submitted to a project-specific website. See text for details.

Figure 3A shows the project-specific web interface and each of the data points on the map indicate a data record (sampling site) submitted via EpiCollect. All samples submitted are listed to the left of the map (Figure 3B) and, using the click-drag-zoom interface, exploration of the data can be undertaken. Clicking one of the sample points displays an ‘info window’ containing the variables collected (soil pH, temp, moisture, GPS position, date etc) and any photograph associated with the record (Figure 3C). Graphical summaries of the variables collected are displayed to the right of the map (Figure 3D) and a number of options are provided for filtering which of the samples points are displayed (Fig 3E). Time sliders allow filtering of data so that only samples collected within a chosen time window are displayed (Figure 3G).

As mentioned, the project-specific curator can communicate with the field workers directly from the web interface using the instant messaging tool, Google Talk. Figure 3F illustrates the chat interface seen by the curator (this is not available in the public view of the website). Chat requires that each submitter has a Gmail account and is sent from the website and automatically received on the phone as seen in Figure 3I. Message transcripts are automatically stored within the user's Gmail account for future reference. Figure 3H illustrates the view using Google Maps of data requested via the phone from the central database and Figure 3J shows the same data viewed at the project website using Google Earth.

Subsequently, the isolates of the bacterial pathogen recovered from the soil samples at each sampling point will be genotyped (by multilocus sequence typing; MLST [1]) and these molecular typing data will be added to the database, allowing further display options, such as the geographic distribution of particular *X. nematophila* genotypes.

### Illustrative project involving multiple field workers

The simple field test illustrated the collection and submission of data from one field worker and the ability to view data retrieved from the central database on the phone using Google Maps. However, EpiCollect is designed for situations where multiple field workers submit data via mobile phone to a central database. For example, multiple field workers may submit data on animal deaths (e.g. surveillance of living and dead amphibians), or animal infections (e.g. using simple field diagnostic tests to distinguish infected and non-infected animals), and the web application allows colour-coding (or symbol-based coding) of the data points submitted by phone to the project database by different field workers.

**Figure 4:**
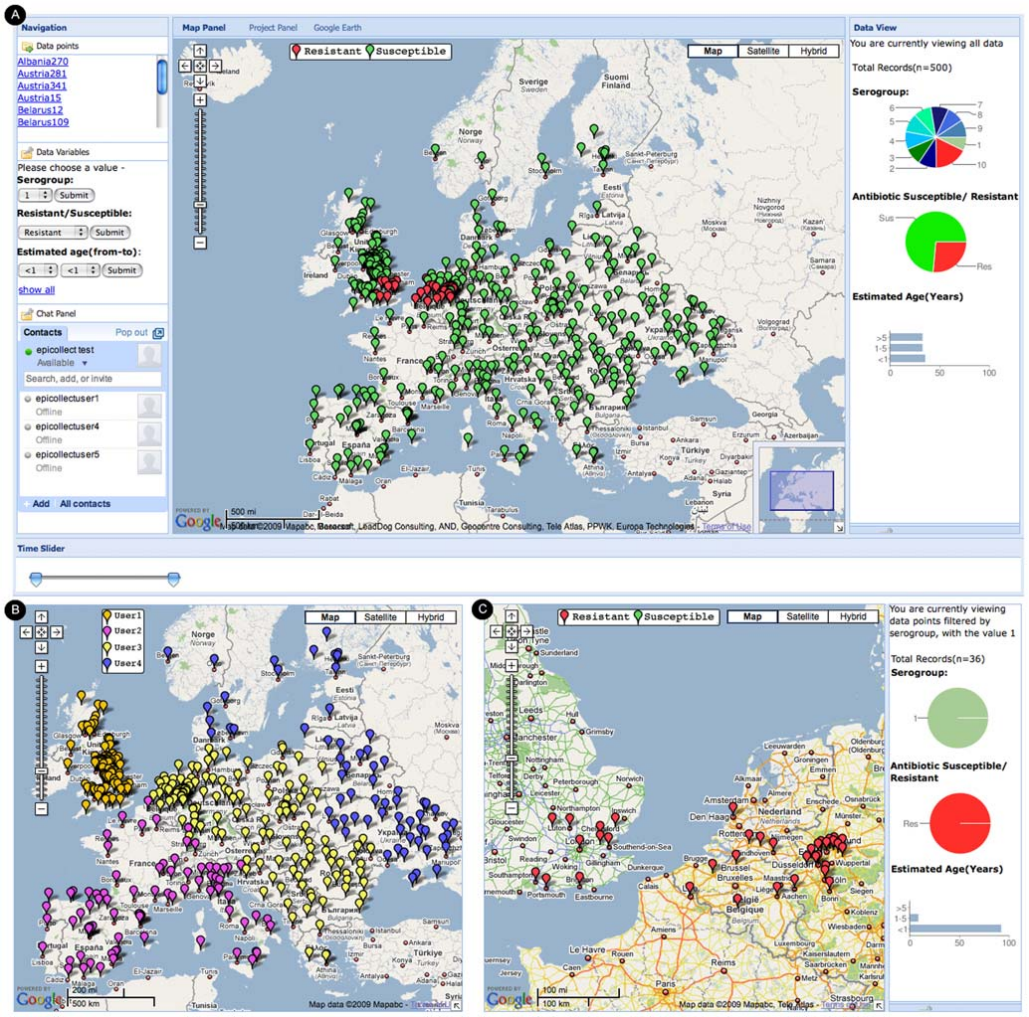
Illustrative dataset showing use of EpiCollect by multiple users. See text for details.

To demonstrate this further, Figure 4 shows the web interface for an illustrative dataset where data from each sampling point have been submitted via mobile phone to the project database by multiple field workers across Europe using EpiCollect. Field tests have been used to identify those animals infected with a specific bacterial pathogen and, as in many real life examples, samples from the infected animals are taken back to a laboratory for strain characterisation (in this case determining the serogroup of the strain and its susceptibility or resistance to an antibiotic). The illustrative data can be explored at http://www.spatialepidemiology.net/epicollect/demo.

Figure 4A shows the overall data summary with each point on the map representing sampling locations across Europe. The points are colour-coded based on whether the pathogen recovered from each of these locations is antibiotic-susceptible (green) or antibiotic-resistant (red). All data points are listed at the top left of the interface and the data variables are graphically represented to the right of the map. The distribution in the total sample of each serogroup, and the extent of resistance to the antibiotic are represented as pie charts, and the estimated ages of the infected animals are represented as a bar chart (<1 year, 1–5 years, >5 years). In Figure 4B the sampling points have been colour-coded based on the individuals who have submitted data from their phones. Using the filtering options shown in Figure 4A we can delimit the data displayed on the map to show only those samples where the animals were infected with serogroup 1 of the pathogen. As seen in Figure 4C, delimiting the data in this way also updates the graphical summaries and shows that all strains of serogroup 1 are antibiotic-resistant and, furthermore, that they are almost always isolated from young animals. In infectious disease epidemiology pathogen isolates are generally characterised at a much finer level than provided by serogrouping using discriminatory molecular typing techniques and, after this has been performed, the geographic distribution and extent of antibiotic resistance of individual genotypes could be displayed on the map. Importantly, these maps can be displayed both on the project website and, by using the filtering options provided by EpiCollect, the same data can be requested and viewed as Google Maps on the mobile phone.

### Community data collection, citizen scientists and further uses of EpiCollect

As the name implies, EpiCollect has been designed to allow the collection of epidemiological data. However, there are many other situations where this kind of two-way communication between mobile phones and a central database could be utilised; for example, biodiversity research, where multiple researchers record details (and photograph) the presence and distribution of a particular species. This approach is exemplified by the Zoological Society of London's EDGE of existence programme which is the only global initiative to focus specifically on threatened species (http://www.edgeofexistence.org).

Increasingly, the potential for ‘citizen scientists’ to collect data for community projects within the biological sciences is being exploited. For example, The Open Air Laboratories (OPAL) network (http://www.opalexplorenature.org) encourages anyone with an interest in nature to explore, study, enjoy and protect their local environment, by undertaking animal and plant surveys (such as earthworms found in gardens) with subsequent web-based data submission. Further examples are provided by FrogLife, which encourages the public submission of back-garden amphibian distributions to aid studies in species decline (http://www.froglife.org), the Evolution MegaLab, which encourages the recording of banding patterns of snails so that members of the public can see ‘evolution in their own back-yards’ (http://www.evolutionmegalab.org) and the RSPB's annual ‘Big Garden Birdwatch’ which requests members of the public to record all species of birdlife seen in back-gardens or parks and submit the data for central collation (http://www.rspb.org.uk/birdwatch). These initiatives involve people recording data in notebooks and then either faxing the data or entering them at the website used for the community project. The use of EpiCollect would allow individuals to directly submit their data to the project database from their mobile phone and could significantly increase the collection and collation of data for these types of community projects, especially amongst the young where the mobile phone is the natural way of transferring information.

We are currently exploring the use of EpiCollect for data submission to molecular epidemiological databases (http://www.mlst.net) and for submissions by field workers to a central database being used to catalogue the world-wide prevalence of the disease caused by the emerging amphibian fungal pathogen, *Batrachochytrium dendrobatidis,* for which we have developed the web application (http://www.spatialepidemiology.net/bd-maps).

### Potential Issues and Further Developments

EpiCollect has been designed for projects that range from epidemiology and ecology to community data collection in developed and middle income countries, but a major potential use is for field surveys in resource-limited settings that collect key information for economic and public health decisions and resource allocation. There are a number of issues centred on cost and mobile data network availability that currently could limit the use of systems such as EpiCollect in these settings – for example in parts of sub-Saharan Africa. GPS satellites, in theory, cover the entire globe and obtaining map co-ordinates for data points entered into a phone using EpiCollect should be possible even in remote areas of resource-limited countries. However, the submission of data from EpiCollect to a central database relies on connectivity with mobile data networks. Mobile phone network coverage in Africa is good in some countries but mobile data networks are much less widely available. The global coverage of such networks can be easily accessed (http://www.gsmworld.com/) and is likely to increase considerably in the next few years. Importantly, data collection and storage within EpiCollect is not reliant on data network connectivity, as data records are stored in the phone's database. Data stored in the phone's database while in remote areas can then be synchronised with the central database after returning to an area that does have data connectivity. However, the major limiting factor for current use of EpiCollect and similar applications in many resource-limited countries is adequate access to data networks and the availability and cost of suitable smartphones. Further progress in the penetration of mobile phone technology may remove these limitations in such areas.

A further general issue is maintaining battery power for mobile phones in remote areas but this can now easily be solved by using solar-powered mobile phone chargers, which are now widely available at low cost.

We chose to develop EpiCollect for Android due to its open-source nature and the provision of suitable multiple phones by different network providers (e.g. T-Mobile, Vodafone and Orange). However, we envision that, similarly to Desktop software that is provided for Windows, Macintosh and Linux, versions will eventually be provided for other smartphone operating systems such as Windows mobile and Palm webOS and an iPhone version of EpiCollect will soon be available.

The generic nature of EpiCollect allows it to be used to submit data to any online database, not just those we host at www.spatialepidemiology.net. We provide EpiCollect as free software and will be further developing the tool to allow on-line project definition of variables for the immediate download of versions of the software tailored to specific needs. We encourage those interested in using EpiCollect within their own projects to contact us, so that we can provide software (and an associated website if required) adapted for use in their projects.

## Materials and Methods

EpiCollect was developed using the Android Operating System SDK. Testing was undertaken using both the developer and commercially available T-Mobile G1 phone running both Android version 1.0 and 1.5. For the project-specific websites located within www.spatialepidemiology.net, interface development utilised the ExtJS, Prototype, Script.aculo.us and Mapstraction JavaScript libraries. We used the Google Maps API for 2D mapping, the KML specification when producing output for Google Earth and the Google Charts API for rendering graphical summaries. Server-side scripting was written in PHP and all data were stored in MS SQL Server.
